# Regulation of Soil Microbial Community Structure and Biomass to Mitigate Soil Greenhouse Gas Emission

**DOI:** 10.3389/fmicb.2022.868862

**Published:** 2022-04-25

**Authors:** Ihsan Muhammad, Ju Zhi Lv, Jun Wang, Shakeel Ahmad, Saqib Farooq, Shamsher Ali, Xun Bo Zhou

**Affiliations:** ^1^Guangxi Colleges and Universities Key Laboratory of Crop Cultivation and Tillage, Agricultural College, Guangxi University, Nanning, China; ^2^Shaanxi Key Laboratory of Earth Surface System and Environmental Carrying Capacity, College of Urban and Environmental Science, Northwest University, Xi’an, China; ^3^Maize Research Institute of Guangxi Academy of Agricultural Sciences, Nanning, China; ^4^Department of Soil and Environment Science, University of Agriculture, Peshawar, Pakistan

**Keywords:** cover crops, soil microbial community structure, greenhouse gas emission, decomposition, cover crop management practices

## Abstract

Sustainable reduction of fertilization with technology acquisition for improving soil quality and realizing green food production is a major strategic demand for global agricultural production. Introducing legume (LCCs) and/or non-legume cover crops (NLCCs) during the fallow period before planting main crops such as wheat and corn increases surface coverage, retains soil moisture content, and absorbs excess mineral nutrients, thus reducing pollution. In addition, the cover crops (CCs) supplement the soil nutrients upon decomposition and have a green manure effect. Compared to the traditional bare land, the introduction of CCs systems has multiple ecological benefits, such as improving soil structure, promoting nutrient cycling, improving soil fertility and microbial activity, controlling soil erosion, and inhibiting weed growth, pests, and diseases. The residual decomposition process of cultivated crops after being pressed into the soil will directly change the soil carbon (C) and nitrogen (N) cycle and greenhouse gas emissions (GHGs), and thus affect the soil microbial activities. This key ecological process determines the realization of various ecological and environmental benefits of the cultivated system. Understanding the mechanism of these ecological environmental benefits provides a scientific basis for the restoration and promotion of cultivated crops in dry farming areas of the world. These findings provide an important contribution for understanding the mutual interrelationships and the research in this area, as well as increasing the use of CCs in the soil for better soil fertility, GHGs mitigation, and improving soil microbial community structure. This literature review studies the effects of crop biomass and quality on soil GHGs emissions, microbial biomass, and community structure of the crop cultivation system, aiming to clarify crop cultivation in theory.

## Introduction

Cover crops (CCs) within agroecosystems impart ecological and environmental benefits, like enhancement of soil fertility, C sequestration, leaching reduction, erosion control, and pest and disease suppression (Alfonso Gomez et al., 2018; Berlanas et al., 2018). Cover crops also increase nutrient cycling and biological N fixation, soil organic matter (SOM), biological diversity (e.g., microbes, insects, and birds), weed control, and crop yields (Muhammad et al., 2019), decreasing drainage, increasing infiltration, and maintaining soil nutrients (Wawan, Dini and Hapsoh., 2019). In addition, CCs provide a friendly agronomic environment with suppression of weeds and thus decreasing the dependency for the herbicides uses (Gavazzi et al., 2010; Barros et al., 2013). Dhima et al. (2006) reported that winter CCs, such as cereal rye (*Secale cereale* L.) and barley (*Hordeum vulgare* L.), could release inhibitory substances known as allelochemicals that can affect the initial growth of grass weeds like barnyard grass.

Previous researchers studied the impact of CCs on SOM in temperate zones and some ephemeral and long-term pools in the Mediterranean and semi-arid annual agroecosystems (Zhou et al., 2016; Austin et al., 2017). Meisinger et al. (1991) reviewed past studies and demonstrated that CCs minimized 20 to 80% of nitrate losses through leaching, whereas NLCCs are more effective than leguminous CCs. They found that winter CCs (small grains) could reduce the nitrate load through leaching and nitrate concentrations by 64 and 50%, respectively. Potential nitrate N leaching in the drainage was minimized by proper crop rotation using CCs (Dinnes et al., 2002). Logsdon et al. (2002) demonstrated that oat and rye CCs significantly decreased nitrate N by 70% in maize-soybean rotation in three simulated years.

Cover crop residues mitigate the negative effects of soil disruption as a result of improving SOM, soil moisture, preventing the germination and emergence of weed seeds, and defending against erosion (Teasdale, 1996; Hall et al., 2010; Alfonso Gomez et al., 2018). Residues mulching maintain the soil moisture by reducing the soil temperature (Unger et al., 1997; Bagley et al., 2012; Li et al., 2013). Cover crops can also affect crop yields through changes in N dynamics in the soil. The addition of NLCCs such as oats tends to reduce mineralization and increases immobilization, lowering the inorganic N availability for the following crop (Haramoto and Brainard, 2012). However, farmers prefer cold-tolerant and productive cereal CCs in the Upper Midwest of the United States and seldom experiment with LCCs (Snapp and Borden, 2005). Leguminous CCs are a rich source of soil N and decompose faster than NLCCs, which results in higher nitrous oxide (N_2_O) emissions, however, NLCCs have a higher C:N ratio and are a rich source of soil organic carbon (SOC), which has higher carbon dioxide (CO_2_) emissions (Muhammad et al., 2019).

**FIGURE 1:**
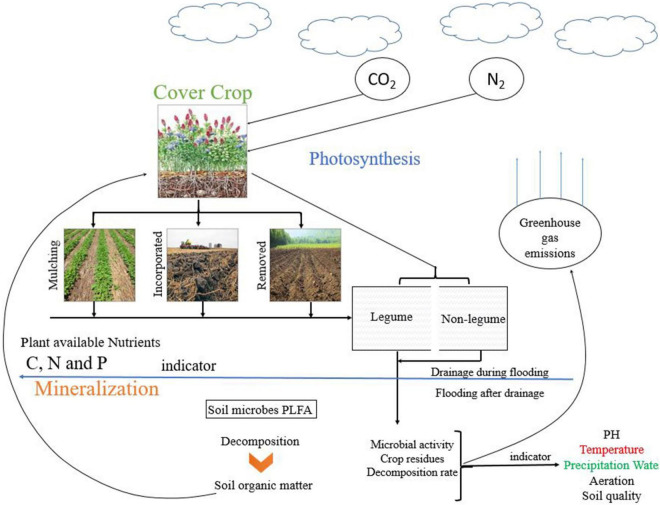
Schematic diagram of CCs growing, termination methods, and their relationship with soil microbes and GHG emissions.

The introduction of CCs into agricultural soil is an important management practice (Figure 1). As shown in Figure 1, the CCs mulching and incorporation increase soil fertility, soil microbial growth, and hence SOM and residues decomposition. The schematic diagram shows that the incorporated residues decomposed faster than mulching, which released more GHGs and rapid availability of nutrients to plants. It has been used extensively to boost SOM and consequently increase cash crop productivity (Sainju and Singh, 2008). CCs cultivation not only provides physical protection to the soil by reducing the impact of rainfall but can also improve soil structure aggregation and microorganisms (Tang et al., 2017; Araujo et al., 2019). Soil organic carbon (SOC) is the critical component of SOM, soil functions, and agricultural ecosystems sustainability (Muhammad et al., 2018; Chalise et al., 2019). Labile organic C is the most active part of SOC and can be used in the short-term experiments as an indicator for assessing the soil quality (Ghimire et al., 2017; Sharma et al., 2018). Furthermore, soil microbes easily access labile organic C and serve as essential nutrients for crop growth (Haynes, 2005). The current review aims (1) to examine the effect of CC types and amount on soil microbial biomass, community abundance and structure, and soil GHGs emissions, (2) to understand the decomposition pattern of CCs residue based on the C:N ratio and its impact on microbial dynamics and GHGs emissions. Excessive fertilization of crops leads to watercourses pollution, thus adding interest to the area of research in green manure practices and its management. Even through a considerable amount of work has been done regarding mineralization, yet the whole concept of residues decomposition, residues quality (low and high C:N ratios), and different CCs types need further detailed exploration in future. Therefore, there is a need to identify the impact of residue management on soil property, soil microbes, and GHGs emission from various residue types and management.

## Manipulating Cover Crop Types to Influence Soil Microbial Biomass, Community, and Greenhouse Gas Emissions

Cover crops have been increasingly grown to improve soil health and crop production and minimize the environmental impact compared to NCCs. It is making a tremendous contribution to the supply of food. CC growing and its residue management is an essential cultural practice for improving productivity on a sustainable basis. This study is truly relevant to the national development and socioeconomic stability of the world. This research will lead to utilizing the organic waste in a better and less harmful manner for crop improvement and soil fertility.

### Legume Cover Crops

**FIGURE 2:**
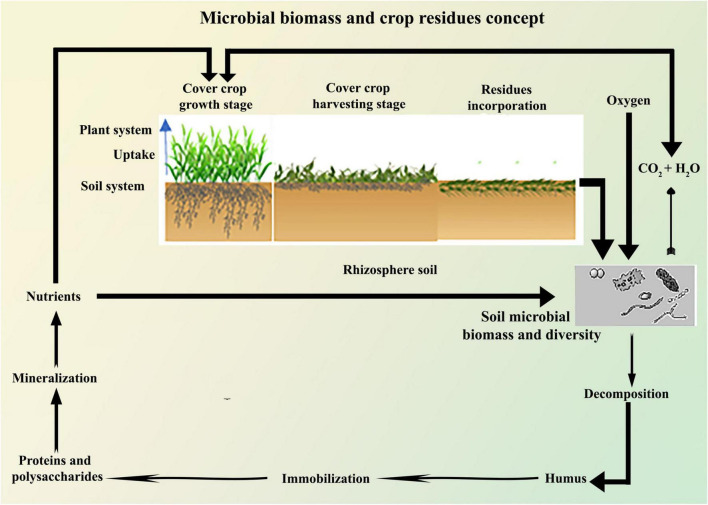
Schematic relationship of residues incorporation, decomposition, nutrient immobilization, and mineralization through soil microbes.

Legume CCs have been used to increase SOM and N concentration (Maltais-Landry and Crews, 2019). The amount of N fixed by legumes is dependent on legume species and environmental conditions (Liebman et al., 2018), and hence increases soil N_2_O emissions (Peyrard et al., 2016). It has been estimated that some LCCs can fix 115 kg of N ha^–1^ year^–1^ from atmospheric N (N_2_) (Peyrard et al., 2016). Kornecki et al. (2016) reported that crimson clover (*Trifolium incarnatum L*.) increased yield by 30% when compared to NCCs plots. However, Reddy (2001) reported that LCCs had reduced the soybean yield when compared to NCC plots. Reddy (2003) observed a 50% reduction in grass weeds, such as barnyard grass, broadleaf signal grass, brown top millet, and a 55% reduction in entire leaf morning-glory (*Ipomoea purpurea*) emergence when using crimson clover. The N taken up by CCs may be subsequently available through mineralization after incorporation (Figure 2), thereby reducing the commercial N fertilizer requirement of the subsequent crop (Ackroyd et al., 2019). The incorporation of LCCs had higher N_2_O emissions than NLCCs and mixed CCs (Peyrard et al., 2016; Kandel et al., 2018). In low input and organic farming systems, the N released after plants incorporation provides a valuable source of N for the following arable crop. In conventional farming systems, CCs have been found to retain up to 60 kg of N ha^–1^ during the growing seasons (De Almeida Acosta et al., 2014). The introduction of LCCs into the soil reduces the inputs of commercial N, thus limiting leaching of N and acts as green manuring (GM) for the succeeding main cash crop (Couedel et al., 2018a; Abdalla et al., 2019). Legumes such as vetch (*Vicia sativa*) and clover (*Trifolium sp*) CCs have higher N fixation capability than NLCCs (Sainju et al., 2007). Legume CCs decompose faster than NLCCs and mixed CCs, which results in higher N_2_O emissions and lower soil CO_2_ emissions (Gonsiorkiewicz Rigon et al., 2018), and thus decreases N leaching and emissions (Muhammad et al., 2019).

Green manuring crops are grown to increase soil fertility and provide a source of N for the subsequent crops. Since GM improves SOM content and especially nutrition value, thus GM is often incorporated in an early immature stage before the cash crop grows (Venkateswarlu et al., 2007; Hwang et al., 2015; Madsen et al., 2016). Legumes are commonly grown as GM due to their high-quality residues (lower C:N ratio) and fixed biological N_2_ from the atmosphere, which leads to decreased N_2_O emissions by 55% (Sanz-Cobena et al., 2014), and decreased N_2_O emissions by 86% in barley crop (Sanz-Cobena et al., 2017). However, CCs during the growth period may reduce gaseous losses by N uptake. Nevertheless, their incorporation may result in increased N_2_O production during nitrification and/or denitrification, released inorganic N in decomposition, and created anaerobic zones in the soil (Baggs et al., 2000; Couedel et al., 2018b). These emissions are generally higher where high N residues such as LCCs are incorporated (Peyrard et al., 2016). The importance of such gaseous losses, in relation to a crop recovery and leaching losses, needs to be quantified to improve N use efficiency in organic systems.

### Non-legume Cover Crops

The capacity of non-legume CCs is to minimize soil erosion (wind and water) and water runoff, increase soil aeration, available water holding, SOM and nutrient cycling (Wawan, Dini and Hapsoh., 2019), reduce NO_3_^–^-N losses in fallow soils, and provide more N for subsequent cash crops (Sainju and Singh, 2008). The impact of NLCCs on N dynamics is not fully understood, particularly in vegetable fields (White et al., 2020). The mineralization of NLCCs by soil microbes can take place on a long fallow period, and readily available mineral N lost as leachate or emissions from fallow (Rodrigues Torres et al., 2008). It was found that CCs can provide 20 to 55% of the recovered N for succeeding crops (Malpassi et al., 2000) beside these losses. However, to reduce the dependency of nitrogenous fertilizers without compromising yield, the mineralization of crop residues N in soil should be synchronized with the N demand of the main crop (Weinert et al., 2002). Cover crop types, growth period, precipitation, and temperature affect N accumulation, N use efficiency, and soil fertility of subsequent cash crops (Toom et al., 2019). Non-legume CCs such as oats and rye produced greater biomass than LCCs (Whitehead and Singh, 2010). Furthermore, grasses use residual N more effectively than LCCs, hence preventing N losses through leaching (Campiglia et al., 2009). Winter CCs are more effective in reducing surface flow and increasing the evapotranspiration of water from field soil. Rotary tillage and rye CCs significantly boosted fungal substrate-induced respiration, SOC, and mean weight diameter (Nakamoto et al., 2012). Similarly, long-term rotations of maize, soybean, rye, and oat CCs decreased concentrations of NO_3_^–^ in tile drainage by 48 and 26%, respectively (Kaspar et al., 2012). Winter CCs are effective, but in the fall, they must grow sufficiently to immobilize residual N in the soil, as shown by Mays et al. (2003), whereas delayed crop growth could be due to late seeding in the fall, which decreases NO_3_^–^ immobilization and increases NO_3_^–^ losses by leaching (Sanz-Cobena et al., 2012).

### Mixed Cover Crops

To increase the utilization of CCs, growers need specific regional information to understand how the biomass and quality of CCs can affect crop yields and reduce emissions. Selection of CC types, tillage practices, termination date, residue decomposition, residue quality, and quantity is highly desirable in such a situation. Residue consistency is often distinguished by the content of C and N, lignification, C:N ratios, and the content of polyphenols (Muhammad et al., 2019; Liu et al., 2021; Wang et al., 2021). If the C:N of crop residues is low, it is generally considered that high-quality crops and nutrients will be released, which affects crop yield strongly and vice versa (Marahatta et al., 2012). A strategy for increasing the quality of crop residues (C:N ratio) and minimizing N immobilization is needed. It has been reported that a mixture of NLCCs and LCCs monocultures is the better choice to improve soil fertility, crop production, and minimize environmental contaminations (Odhiambo and Bomke, 2000). Mixed CCs provide another strategy to mitigate environmental problems because they have a relatively high C:N ratio compared with LCCs and consequently reduce N_2_O emissions (Aita and Giacomini, 2003; Schmeer et al., 2014).

## Cover Crop Uses and Benefits for Soil Microbial Biomass and Community Improvement

Cover crop cultivation and its residues management practices are the main factors that improve soil water holding capacity, soil microbial abundance and structure, and weed suppression. CCs incorporation, mulching, and removal from the field after harvesting have a critical impact on soil microorganisms. The influence of Cover crop types, residues management, and restudies quality are the key strategies to improve soil microbial communities, soil bacteria, and soil fungi.

### Soil Microbes

Decades of intensive farming have reduced SOM content, thus plummeting soil fertility and arable land biodiversity (Gardiano et al., 2013). Subsequently, important services for soil ecosystems like nutrient cycling, water management, C storage, and functional biodiversity have been impaired. Microbial communities are vital for improving soil structure conservation and act as main decomposers of fresh organic material and drive biogeochemical nutrients transformation (Pina, 2019). The impacts of management practices on microbial populations are well known, at least regarding the increase in bacterial abundance and enzymatic activity (Muhammad et al., 2021a). Soil with sweet corn residue removed, incorporated, or garland chrysanthemum had 5.0, 5.4, and 6.2% higher microbial populations and 22, 32, and 26% higher fruit yield, respectively, than control soil (Tian et al., 2011). Similarly, the perennial CCs increased the N mineralization rate and MBC by 37 and 41%, respectively, compared to the NCCs (Pandey and Begum, 2010). In organic farming, huge amounts of C are usually incorporated into the soil, replacing mineral fertilizers ultimately increases the SOM content (Lal, 2009). In a recent study, the SOM content was increased in organic farming as compared to non-organic farming (Cagnini et al., 2019). The introduction of CCs increased the quantity of SOC and improved SOM, microbial biomass carbon, and microbial community structure (Finney et al., 2017). These modifications are essentially based on the characteristics of CCs chemistry and the biotic interactions between plant and soil. Leguminous CCs can fix more atmospheric N due to rhizobia increasing the mineralization and N pool of soil (Schroth et al., 2001). Similarly, previous studies have shown that different CC species had strong correlations with soil microbial biomass, suggesting that milk vetch had the highest microbial biomass N (15.4 mg kg^–1^) followed by ryegrass (11.3 mg kg^–1^), while the lowest 6.1 mg kg^–1^ was observed for the NCCs (Zhu et al., 2012).

**FIGURE 3:**
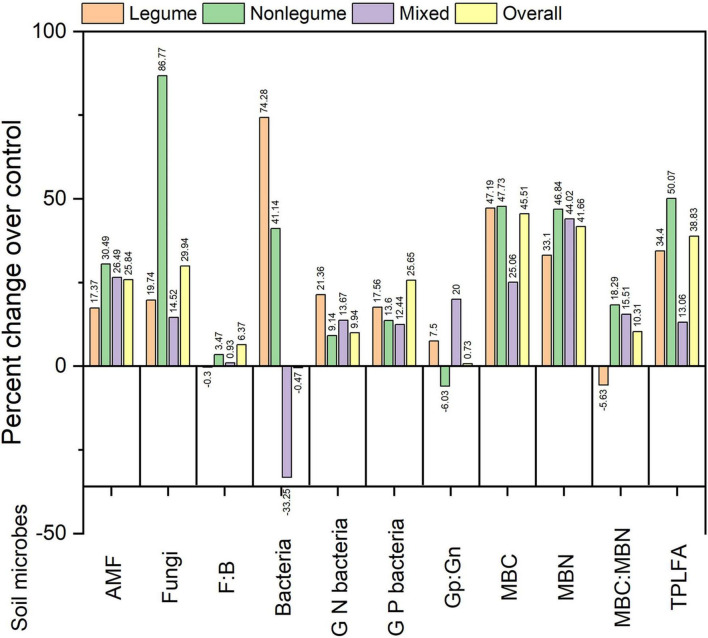
Percent changes of AMF, fungi, F:B, bacteria, Gram-positive bacteria (G P bacteria), Gram-negative bacteria (G N bacteria), Gram positive: Gram negative bacteria (Gp:Gn), microbial biomass carbon (MBC), microbial biomass nitrogen (MBN), MBC:MBN, and total phospholipid fatty acid analysis (PLFA) in cover crop (CC) treatments over control (Ncc).

Growing CCs create a conducive environment for microbial growth and activities, and upon the decay of CCs, the fungi are attacking residues first followed by bacteria (Hodge et al., 2001). CCs had a positive impact on soil microbial abundance, microbial activity, plant metabolism affecting soil respiration, and plant mineral nutrition (Setyawan et al., 2011), depending on the weather, plant species, and the season of the year. Grasses as CCs played an important role in soil management in citrus orchards and showed that grass roots release stimulating compounds for arbuscular mycorrhizal fungi, which is beneficial for plant growth (Lekberg and Koide, 2005). Legume CCs are more effective at fixing N, which is necessary for protein synthesis and plant growth. According to our estimation, the LCCs increase AMF, fungi, bacteria, MBC, MBN, and total PLFA by 17.37, 19.74, 74.2847.19, 33.1, and 34.4% compared to NCCs, respectively (Figure 3), however, fungi to bacteria ratio (F:B) and MBC:MBN ratio decreased by 0.3 and 5.63%, respectively. These results are in line with the finding of Cavalca et al. (2013), who reported that the CCs increase SOM content and microbial activity. The cultivation of CCs adds both the above-ground and below-ground biomass to soil (Wang et al., 2010), which raised the soil C and N stocks (Sainju et al., 2006; Buechi et al., 2015). Growing CCs are encouraged to optimize the productive use of N for a subsequent cash crop and increase productivity due to decreased nutrient losses through leaching (Valkama et al., 2016). Furthermore, reducing dependency on mineral fertilizers improved water holding capacity, suppressing insect pests and weeds (Dorn et al., 2015; Brooks et al., 2018; Maltais-Landry and Crews, 2019).

### Microbial Communities

The introduction of CCs into the agricultural system improves soil and environmental quality through increasing soil microbial population and reducing the application of chemical fertilization (Mitchell et al., 2017). Blanco-Canqui and Jasa (2019) testified that CCs enhanced the soil chemical, physical, and biological properties. CC utilization reduces soil erosion by covering the soil and improves soil quality by cycling nutrients, SOM, and MBC (Kerri and Belina, 2008). CCs significantly impact microbial communities’ size, operation, and structure by increasing soil C inputs (Xi et al., 2010). Soil microorganisms play a significant role in soil feeding, development, and restoration (Calderón et al., 2016; Zhou et al., 2017). According to Peregrina et al. (2014), soil microbial population characteristics are strongly linked to microbial biodiversity, soil and plant quality, and ecosystem sustainability. Researchers argued that nutrient cycling and C conservation are driven by soil microbial communities and vegetation species diversity (Carney and Matson, 2006). Soil microorganisms release enzymes that facilitate the breakdown of complex components in organic materials, and correlate enzymes with soil organic C and N substances, which is an indicators of microbial community due to changes in management systems (Tian et al., 2010).

Cover crops significantly improve SOM and boost soil microbial communities (Bacq-Labreuil et al., 2019). In addition, CCs used as GM could boost soil AMF, bacteria, microbial biomass, and total PLFA compared to NCCs (Figure 3). Higo et al. (2018) concluded that LCCs in rotation (corn-legume) form a symbiotic relationship with particular mycorrhizal fungi and bacterial groups. The continuous crop rotations are collectively referred to as legacy effects, such as beneficial legacy impacts enhancing competent AMF richness, AMF spore density, AMF root colonization, and microbial diversity, eventually improving soil health and agricultural production (Higo et al., 2019). Nonetheless, some of these legacy effects may not be necessary, such as replacing capable AMF with non-host crops and escalating the potential for nutrient immobilization and mineralization in subsurface soil in winter-wheat (Somenahally et al., 2018).

### Soil Bacteria

The maintenance of biological health is important for restoring deteriorated soil because the living components of the soil are necessary for ecosystem functions and utilities (Lehman et al., 2015). CCs rotation and minimal tillage are approaches for enhancing the sequestration of organic compounds in agroecosystems that seem to be the most significant ecosystem services and significantly impact the soil biota. It was reported that the first principal component (PC1) distinguished the vetch treatments from the NCCs and wheat treatments, accounting for 23.4% of the total variability (Mbuthia et al., 2015). Gram-positive bacteria (i17:0, i16:0, a15:0, a17:0), Gram-negative bacteria (cy19:0ω8c,16.1ω7c), and actinomycetes (10Me16:0, 10Me17:0, 10Me18:0) were found in greater abundance in communities under the vetch CCs treatment. Communities with NCCs and wheat treatments, on the other hand, were linked to the mycorrhizae fungi fatty acid methyl ester biomarker (18:1ω9c) and the saprophytic fungi biomarker (18:2ω6c; Mbuthia et al., 2015). Cover crops and biological fertilizers are critical aspects of soil quality and fertility in organic management systems. Researchers discovered that CCs prevent soil C, boost SOM, reduce nutrient leaching, and LCCs fix N biologically (Snapp and Borden, 2005). CCs influence below ground soil functioning through soil microbial communities, such as nutrient cycling and availability, decomposition and transformation of crop residues, and disease suppression (Garbeva et al., 2004; Van Der Heijden et al., 2008). Cover crops generally increase overall microbial activity, nutrient cycling, and microbial diversity (Lori et al., 2017). Similarly, different compositions of CCs cause various changes in soil microbial populations, such as microbial infection, Gram-positive bacteria, Gram-negative bacteria, and bacterial/fungal ratios (Wortman et al., 2013; Buyer et al., 2017). It was reported that calopo (*Calopogonium mucunoides*) and callisia (*Callisia repens*) residues have 30 and 25% higher AMF than NCCs, respectively. However, mixed CCs significantly increased all the soil microbial biomass and community structure but decreased the soil bacteria by 33.25% (Figure 3). A previous study reported that root C content is more critical than residues C in maintaining stable C (Kong and Six, 2012) suggesting that the effect of litter quality on the sequestration of C is crucial (Mueller et al., 2017). Frasier et al. (2016) demonstrated that high quality litter and more effective soil biota would improve SOC stability and ultimately increase its storage.

**FIGURE 4:**
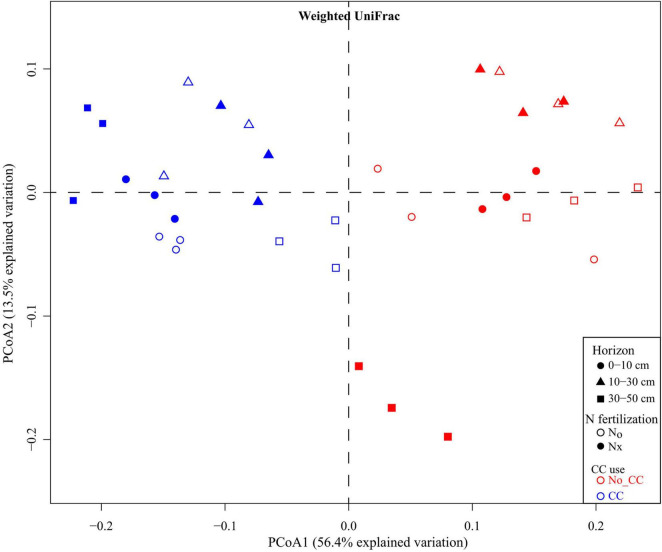
Principal coordinates analysis (PCoA) of microbial communities based on weighted UniFrac distances across soil horizons and among the different experimental treatments (Nx: conventional N fertilization; N0: no N fertilization; CC: presence of cover crops; No-CC: bare soil conditions). (Copied with permission from Alahmad et al., 2019, copyright (2018) John Wiley & Sons, Inc).

Cover crops, the most commonly grown vegetation between cash crops, serve as organic matter modifications to the agroecosystems and increase SOC (Sharma et al., 2018). Studies have confirmed that CCs increased SOM and nutrient use efficiency in agroecosystems by reducing nitrate N losses through leaching and drainage, thereby increasing soil bacteria (Rice and Gowda, 2011; Alahmad et al., 2019). According to the microbial community structure estimated by PLFA profiles, the total bacterial and Gram-positive bacteria were significantly higher in CCs than in NCCs treatments. The PCoA analysis showed that the first two principal components, PC1 and PC2, accounted for 13.5 and 56.4% of the total bacterial variability. According to the PCoA, bacterial communities were clearly clustered according to their utilization of CCs (Figure 4), suggesting that the PC2 clearly separated the bacterial communities of CCs and NCCs plots. Furthermore, the bacterial community was not significantly affected by CCs with N fertilization in 10–30 cm soil depth (Alahmad et al., 2019); however, it reduced wind and water erosion (Moreira Rovedder and Foletto Eltz, 2008). The production of CCs improved soil quality and soil biota (Morales Salmeron et al., 2019). CC residues increased the amount of labile C in the agroecosystem, especially in the spring season after harvest, and the primary consumer of this labile C is the microbial community (Fernandez et al., 2016). These results show that glucose is a vital source of energy in microbial metabolism (Mukumbareza et al., 2016).

**FIGURE 5:**
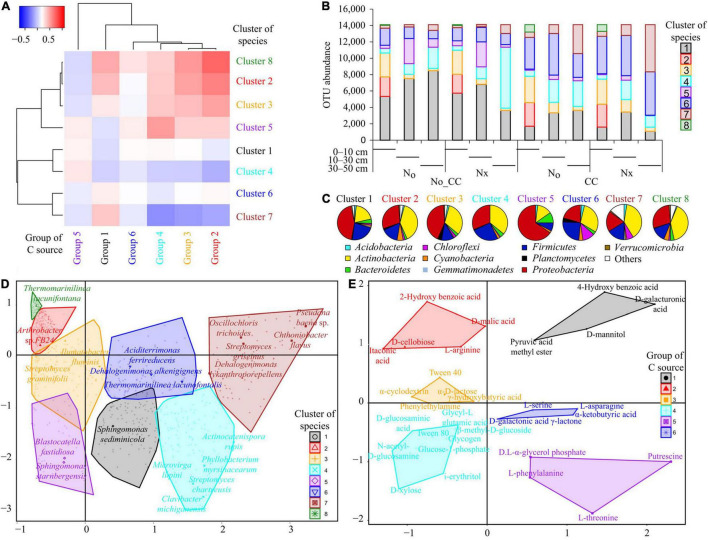
Results of the hierarchical clustering of bacterial species composition and used C-sources. **(A)** Heat-map of C-source groups and species clusters based on their values in the cross-table of the BGCoIA. **(B)** Relative importance of the clusters across soil depths and among treatments (Nx: conventional N fertilization; N0: no N fertilization; CC: presence of cover crops; No_CC: bare soil conditions). **(C)** Distribution of the main bacterial phyla among clusters. **(D)** Projection of the clusters and their constitutive species in the diagram defined by the first two BGCoIA axes. Only the name of the most characteristic species for each cluster is reported. **(E)** Projection of the groups and their constitutive C sources in the diagram defined by the first two BGCoIA axes. (Copied with permission from Alahmad et al., 2019, copyright (2018) John Wiley & Sons, Inc).

It was observed that cluster 5 (Figure 5) was associated with NCCs, dominated by Proteobacteria, and characterized by *Blastocatella fastidiosa* and *Sphingomonas starnbergensis*. The CCs were linked to Cluster 6, which included species from all major and minor phyla and was as described by *Aciditerrimonas ferrireducens* and *Dehalogenimonas alkenigignensi* (Figure 5). In CCs, the community was dominated by Actinobacteria, with *Oscillochloris trichoides* and *Streptomyces griseus* as characteristic species and containing many species from “other phyla” (Alahmad et al., 2019). The community in NCCs was characterized by species from Cluster 4, which included Proteobacteria, Actinobacteria, and Firmicutes, with *Actinocatenispora rupis* and *Streptomyces chartreusis* as representative species. Nitrogen fertilization resulted in functional divergence regardless of CCs, but this was supported by compositional divergence only in the presence of NCCs (Figure 5).

### Soil Fungi

Kingdom fungi include a morphologically diverse group of species extending from single lad yeast to macro fungi, forming networks in soil over many meters. Fungi have attracted attention as major crop pathogens in cultivated agriculture. Nevertheless, they also play a key role in nutrient cycling via dead organic matter catabolism and mycorrhizal symbionts (Chavarria et al., 2016). In cultivated and grassy soils, AMF such as Glomeromycota is the primary mycorrhizal symbiont. Mycorrhizal colonization increased by 35, 29.4, and 20.9%, with hairy vetch, mixed CCs, and Indian mustard in maize crops, respectively. This suggests that releasing isothiocyanates in soil resulted in higher shoot biomass, N, and phosphorus content across all maize genotypes with mycorrhizal colonization (Njeru et al., 2013). However, recent progress in plant-soil interactions suggests that fungi have a broader range of effects that interact with higher grassland plants and thus play an important role in plant nutrition (Higo et al., 2017). CCs minimized nitrate leaching and plant disease and increased microbial populations and community structure (Singh and Kumar, 2021). White clover crop mulching increased AMF colonization and maize production (Deguchi et al., 2012). It has also been stated that the introduction of CCs during the bare fallow season increases the AMF inoculum capacity, AMF colonization, and production of subsequently cultivated major crops (Hontoria et al., 2019). Canonical correspondence analysis showed the relationships between AMF communities and winter CCs, revealing that winter CCs had a significant impact (*F* = 3.187, *P* = 0.001) on AMF communities (Higo et al., 2015).

Arbuscular mycorrhizal fungi provide many advantages in the symbiosis process for most plant species among various classes of microorganisms. Smith and Read (2008) suggested that AMF offered numerous benefits for host plants, including enhanced nutrition uptake, particularly in poor nutrient soils and plants tolerance to biotic and abiotic stresses. The AMF significantly improves agroecosystems sustainability and productibility while simultaneously decreasing the use of synthetic fertilizers (Lehman et al., 2015). Cover crops in agricultural practices permit native inoculum recovery and biodiversity, which are of particular interest to low-input systems (Martinez-Garcia et al., 2018). According to a structural equation model, root colonization (λ = 1.149) and maize phosphorus uptake (λ = 1.185) had immediate strong positive effects on crop performance, whereas AMF diversity (λ = 0.395) had intermediate positive effects (Higo et al., 2018). A recent study has shown that replacing fallow with CCs during intercropping time improves AMF root colonization in the succeeding cash crop (Cagnini et al., 2019).

**FIGURE 6:**
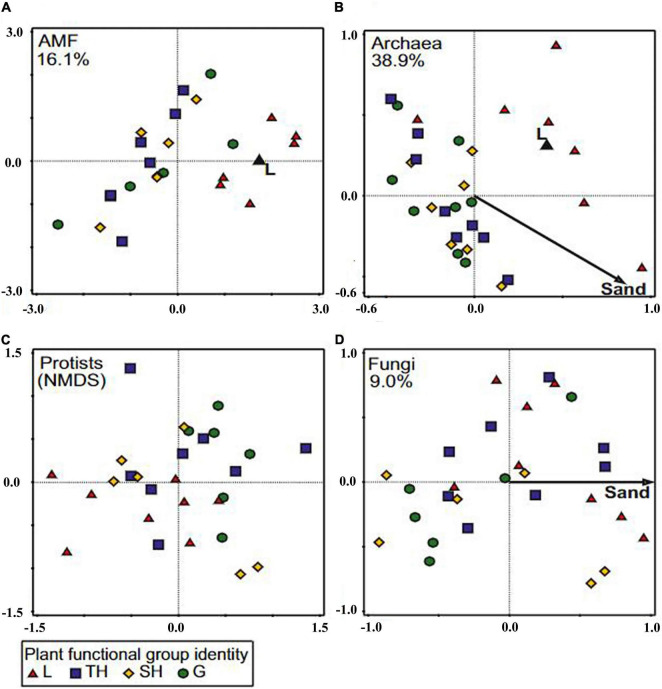
Distance-based redundancy analysis (db-RDA) plot showing the relationship of abiotic soil factors and plant functional group identities to community composition of AMF **(A)**, archaea **(B)**, and fungi **(D)**. Community composition of protists **(C)** could not be explained by any of the factors measured; therefore, a non-metric multidimensional scaling (NMDS) of the community is shown instead. The plant functional groups tested were grasses (G; green), legumes (L; red), small herbs (SH; yellow), and tall herbs (TH; blue). The ordination is based on Bray–Curtis distance. With forward selection, factors were chosen that significantly (*P*_*adj*_ < 0.05) contributed to the model. In each window, the percentage of explained variation is shown. (Adapted from Dassen et al., 2017 under the terms of the Creative Commons Attribution License 4.0).

Cover crop with negligible or zero tillage facilitate mycelium and make colonization faster (Brito et al., 2012). Likewise, CCs improve nutrient reusability, regulating weed growth, reducing erosion, subduing soil disease, and decreasing nutrient leakage (Briar et al., 2011). However, a limited number of literature studies are published to elaborate CCs effect on the AMF community (Bowles et al., 2017). Previous studies suggested that winter CCs influence and change the structure of the AMF population in the subsequent cash crops (Higo et al., 2018; Morimoto et al., 2018). However, instead of long-term experiments, researchers mostly studied short- to mid-term experiments in the past. Therefore, a literature review on how CC types and management practices influence the operation and microbial communities is required. Blanco-Canqui and Jasa (2019) reported that the AMF population could be significantly affected by various environmental factors, such as soil properties, nutrient availability, and air temperature. Arbuscular mycorrhizal fungi communities in legume CC plots are different from those in grass and herb plots (Figure 6A) and explain 16.1% of the variation in AMF communities. Likewise, archaeal communities in legume plots differed from grass and herb plots (Figure 6B), and the plant functional group accounted for 14.0% of the total variation. Regarding protists community composition, plant functional group had no significant explanatory power (Figure 6C). Abiotic properties appeared important for fungi and archaea, as shown in Figure 6D. The abiotic properties seemed important for fungal and archaea communities, which explained 9.0% of fungal community composition and 24.9% of archaeal community composition (Dassen et al., 2017).

## Role of Soil Microbes in Greenhouse Gas Emissions

Crop production is the focusing center of agriculture in the world. It is making a tremendous contribution to the supply of food. CC growing and its residue management is an essential cultural practice for improving productivity and decreasing GHGs on a sustainable basis.

### Greenhouse Gas Emissions

Agricultural soils are supposed to be a significant source of greenhouse gas (GHG) emissions, primarily nitrous oxide (N_2_O) and methane (CH_4_) as reported (IPCC, 2007). Over the past 100 years, the average global surface temperature has increased by 0.3–0.6°C due to the increase in GHG emissions (He et al., 2009). Carbon dioxide, N_2_O, and CH_4_ are the three main GHGs, and the increase in CO_2_ concentration contributes the most to the greenhouse effect (Ozturk and Acaravci, 2010). GHG emissions due to agricultural farming account for 10–12% of total GHG emissions. Agriculture is a large-scale human activity and has an important impact on global greenhouse emissions (Shelton et al., 2017). Numerous agricultural activities (e.g., irrigation, N fertilization, residues management) have been found to drive variables for these gas emissions during the crop cycle (IPCC, 2007; Muhammad et al., 2022). Some of these activities often impact gas emissions during the fallow period, particularly crops with low N use efficiency (NUE), while significant quantities of mineral N remain in the soil after the harvest (Sanchez et al., 2019). Intensively irrigated and fertilized agricultural systems generally have high levels of inorganic N that are lost in the ecosystem via leaching or denitrification during fallow or crop periods (Sanz-Cobena et al., 2012). Replacing bare fallow with CCs in the agricultural system is one of the most agronomic strategies for increasing the retention of inorganic surplus N and reducing nutrient losses (nitrate) through leaching (Dinnes et al., 2002).

In Mediterranean climate zones, winter CCs are used as catch crops (Gabriel and Quemada, 2011). However, due to CCs, the N and water requirements change and C pools may affect the processes leading to GHG emissions. CC root depth, crop N, water requirements, rhizosphere nutrient release, and climate change adaption are strategic factors for mitigating GHGs (Gabriel and Quemada, 2011). In this context, cereals crops generally reduce soil N content in the early growth stages due to higher N uptake and reduced losses through drainage water (Thorup-Kristensen et al., 2003; Moeller et al., 2008). Whereas cruciferous plants have a greater capacity to absorb N in deeper rooting systems at later growth stages and are more easily extracted from the deeper areas (Thorup-Kristensen et al., 2003). Legumes were assessed as catch crops, and it was shown that 50 to 60% of the N in legume tissue mainly comes from N absorption (Pappa et al., 2011). The soil alterations that affect GHGs during the CCs formation and its relation with GHGs are limited (Bayer et al., 2016).

After harvesting, CCs biomass is typically used as GM to reuse the N reserved in residues biomass and contribute to sustainable long-term soil fertility (Chirinda et al., 2010). The quality of CCs is mainly dependent on the C:N ratio, crop lignin content, and the N fixing capability, which affect the dynamics of C and N (Sofi et al., 2018). Incorporating crop residues into fertile soils will increase N_2_O emissions from cultivated soils (Bair et al., 2008), which depends largely on the residual biomass composition, suggesting that residues with low C:N ratios generally increase N_2_O emissions (Huang et al., 2016). Temporary N fixation often reduces N_2_O loss, and thus delays N availability for the crops as a result of CCs (Marahatta et al., 2012). In crop rotation, the N use efficiency of the main crops (cereals) is affected by GM of previous CCs and other N sources such as fertilizer. The joint effects of residues and synthetic N sources will also affect N_2_O emissions. Mineral N in a fertilized plot with greater C:N ratio residues promotes and increases N_2_O and CO_2_ emissions compared to sole N fertilizer because soluble residual organic C can be used as energy for denitrification (Ghimire et al., 2017; Ozturk, 2017). In terms of CH_4_ emissions, the amount of mineral N in the soil may influence the oxidation of this compound (Sanz-Cobena et al., 2014). The higher emissions might be due to the mineral N pool increasing through mineralization of N-rich plant material from legumes used as GM. Specific GHG emissions enable us to recommend specific crop management methods in irrigated corn systems to mitigate these losses (Rosolem et al., 2004; Sogbedji et al., 2006). At present, there are many studies on the effects of CC types and management practices on soil GHG emissions, but limited studies are available on CCs biomass rates and residue C:N ratios. The characteristics of GHGs emissions after CCs harvesting and whether the management of the residues (e.g., mulch, incorporation, and removal) are unclear. Thus, this review documented the effect of residue quality and quantity on different GHGs under different climatic reigns.

### Carbon Dioxide Emissions

Greenhouse gas emissions from agricultural soils are affected by a variety of factors. After CCs introduction to the soil, the C and N cycle, pH, and biomass (microorganisms, roots, etc.) change, which affects GHG emissions in agriculture soil. Behnke and Villamil (2019) reported that CC introductions to the soil increased GHG emissions, which affected the initial C sequestration. In contrast, CCs mulching effectively reduced the GHG emissions (Tribouillois et al., 2018). CCs may accelerate soil CO_2_ efflux compared to conventional farming by increasing SOC and microbiological properties at the 0 to 2.5 cm soil surface (Peregrina, 2016). CC mulching increases the total porosity of the soil, and increases CO_2_ content of soil solution, which is conducive to the diffusion of CO_2_ (Mondal and Lenka, 2012). The range of CO_2_ emissions were 158 to 1884 and 154 to 1613 mg kg^–1^ for surface-applied and incorporated residues, respectively, and the highest emissions were found under the clover crop while the lowest were for the fallow (Linsler et al., 2016).

To use CCs, farmers select species that fulfill two functions: to keep soil protection and fertility in balance. Xiao et al. (2013) reported that CCs increased SOC content and changed it to a water-soluble form in the soil, thereby affecting the CO_2_ emission mode. Compared to NCC, the winter LCCs in the growing season had significantly higher CO_2_ emissions than in the rainy season. The range between NCCs (96.92 to 259.05 mg CO_2_ m^–2^ h^–1^) and winter LCCs (106.28 to 353.01 mg CO_2_ m^–2^ h^–1^) are shown in Table 1. Common vetch as a GM resulted in lowering soil C:N and higher CO_2_ emissions (range of CO_2_ 8.09 to 10.58 g CO_2_ m^–2^ h^–1^), enzyme activity, and N release. Conversely, the GM of rye grass had a slightly lower CO_2_ emission than vetch CCs (range of CO_2_ emissions; 6.63 to 8.19 g CO_2_ m^–2^ h^–1^) and early soil N immobilization (Table 1). In contrast, NLCCs decreased CO_2_ emissions. Winter LCCs treatment had the greatest effect on soil CO_2_ and N_2_O emissions under furrow irrigation (Kallenbach et al., 2010). Zhou et al. (2019) suggested that CCs provide a C source for microorganisms, which promotes microbial growth and metabolism, thereby increasing CO_2_ emissions. A researcher demonstrated that CCs incorporation increases the content of water-stable aggregates in the surface soil, which positively correlated with CO_2_ emissions (Uliarte et al., 2013). In addition, CCs mulching can indirectly affect soil CO_2_ emissions by changing conditions such as preventing soil water losses and maintaining soil temperature. Kokalis-Burelle et al. (2017) reported that mulching of sun hemp as a CC improves the living environment of soil microorganisms increases microbial activity and may lead to increased CO_2_ emissions. Researchers found that CO_2_ emissions from bare land were much higher than those of CCs treatment, mainly because mulching reduced surface soil temperature and prevented the soil from emitting CO_2_ into the atmosphere (Li et al., 2013; Liang et al., 2017).

**TABLE 1:** Role of different cover crop types (legume, non-legume, and mixed) in soil carbon dioxide emissions (CO_2_).

Author	Soil-types	Vegetation	Cover crop	CC type	Range of CO_2_ emissions		Units
					Control	Cover crop	
Kallenbach et al., 2010	Coarse-loamy	Tomato	Winter legume	Legume	259.05	307.66	mg CO_2_ m^–2^ h^–1^
Kallenbach et al., 2010	Coarse-loamy	Tomato	Winter legume	Legume	215.36	353.01	mg CO_2_ m^–2^ h^–1^
Kallenbach et al., 2010	Coarse-loamy	Tomato	Winter legume	Legume	96.92	106.29	mg CO_2_ m^–2^ h^–1^
Kallenbach et al., 2010	Coarse-loamy	Tomato	Winter legume	Legume	106.07	162.22	mg CO_2_ m^–2^ h^–1^
Mancinelli et al., 2013	Sandy loam	Sweet pepper	Rye grass	Non-legume	5.17 to 6.53	6.63 to 8.19	g CO_2_ m^–2^ h^–1^
Mancinelli et al., 2013	Sandy loam	Sweet pepper	Common vetch	Legume	5.17 to 6.53	8.09 to 10.58	g CO_2_ m^–2^ h^–1^
Uliarte et al., 2013	Clay/silt	Vineyards	*Festuca arundinacea*	Non-legume	0.28 to 0.58	–1.4738	g CO_2_ m^–2^ h^–1^
Sanz-Cobena et al., 2014	Silty clay loam	Maize	Barley	Non-legume	2.71 to 9.01	5.16 to 16.54	kg CO_2_-C ha^–1^ d^–1^
Sanz-Cobena et al., 2014	Silty clay loam	Maize	Rape	Non-legume	2.71 to 9.01	3.97 to 12.28	kg CO_2_-C ha^–1^ d^–1^
Sanz-Cobena et al., 2014	Silty clay loam	Maize	Vetch	Legume	2.71 - 9.01	2.98 to 16.06	kg CO_2_-C ha^–1^ d^–1^
Bavin et al., 2009	Silt loam	Soybean	Rye	Non-legume	2.64 to 4.06	3.23 to 3.68	μmol m^–2^ s^–1^
Negassa et al., 2015	Fine loamy	Maize/soybean	Rye	Non-legume	73.03 to 136.18	87.17 to 151.73	mg CO_2_ m^–2^ h^–1^
Hwang et al., 2017	Clay loam	Rice	Barley	Non-legume	72.62 to 172.81	188.33 to 219.04	mg CO_2_ m^–2^ h^–1^
Hwang et al., 2017	Clay loam	Rice	Hairy Vetch	Legume	72.62 to 172.81	173.32 to 274.31	mg CO_2_ m^–2^ h^–1^
Hwang et al., 2017	Clay loam	Rice	Barley + Vetch	Mixed	72.62 to 172.81	200.43 to 297.24	mg CO_2_ m^–2^ h^–1^
Abdalla et al., 2014	Sandy loam	Spring barley	Mustered	Non-legume	24.51	26.09	mg CO_2_-C m^–2^ h^–1^
Xavier et al., 2013	Sandy loam	Dwarf cashew	Butterfly pea and pigeon pea	Legume	7.47 to 5.54	7.47 to 8.57	mg CO_2_-C kg^–1^ soil day^–1^
Peregrina, 2016	Fine-loamy	Vitis vinifera L. Vineyard	Resident vegetation	Non-legume	0.231 to 0.231	0.41 to 0.45	μmole CO_2_- m^–2^ S^–1^
Steenwerth and Belina, 2008	Coarse–loamy	Vineyard	Rye	Non-legume	2.5 to 13.41	6.47 to 16.2	μg CO_2_-C m^–2^ S^–1^
Steenwerth and Belina, 2008	Coarse–loamy	Vineyard	Trios	Non-legume	2.50 to 13.41	4.37 to 22.92	μg CO_2_-C m^–2^ S^–1^
Barrios-Masias et al., 2011	Silt loam	Tomato	Winter mustard	Non-legume	52.17 to 151.63	71.73 to 228.26	mg CO_2_-C m^–2^ h^–1^
Rosecrance and Teasdale, 2000	Coarse-loamy	Maize	Rye	Non-legume	1.37 to 59.32	4.99 to 544.36	mg core^–1^
Rosecrance and Teasdale, 2000	Coarse-loamy	Maize	Vetch	Legume	1.37 to 59.32	9.99 to 558.60	mg core^–1^
Rosecrance and Teasdale, 2000	Coarse-loamy	Maize	Rye + Vetch	Mixed	1.37 to 59.32	3.84 to 552.71	mg core^–1^
Bhattacharyya et al., 2012	Sandy clay loam	Rice	*Sesbania aculeata*	Legume	14.80 to 35.25	14.81 to 68.06	mg m^–2^ h^–1^
Liebig et al., 2010	Silt loam	Wheat	Rye	Non-legume	5.52 to 45.71	8.23 to 49.80	mg CO_2_-C m^–2^ h^–1^
Baggs et al., 2003	Silt loam soil	Maize	Rye	Non-legume	194 to 490	132 to 937	kg CO_2_-C ha^–1^
Baggs et al., 2003	Silt loam soil	Maize	Bean	Legume	194 to 490	314 to 901	kg CO_2_-C ha^–1^

### Nitrous Oxide Emissions

Cover crop will minimize N losses from agricultural practices by minimizing both nitrate leaching and ammonia plus nitrous oxide transport to the atmosphere (Lacey and Armstrong, 2015). Nitrous oxide is the major contributor to global warming from the agricultural farming system (IPCC, 2007), which is released in soils primarily through two coupled microbial processes; nitrification in aerobic and denitrification in anaerobic environments (Bowen et al., 2018). The frequency and strength of these procedures are strongly influenced by the availability of soil mineral N, soluble C, water, oxygen, temperature, pH, and soil texture (Li et al., 2019). As changes in farming techniques directly affect substrate quality and the environmental conditions in the soil, they are expected to have an effect on N_2_O emissions (Abdalla et al., 2012). Steenwerth and Belina (2008) demonstrated that legume CCs might lead to greater N_2_O emissions, and much of this increase could be due to the decomposition of crop residues. The global impact of legume CCs during crop rotation on N_2_O emissions tends to be mostly neutral, although it may differ in relation to the quality (C:N ratios) of crop residues and management techniques (Muhammad et al., 2019). The seasonal N_2_O flux in barley cultivation did not differ from that in the NCCs (3.6 kg ha^–1^). However, LCCs (hairy vetch and/or barley + hairy vetch mixture) treatments increased seasonal N_2_O flux by 1.8 times than the barley treatment alone (Table 2).

**TABLE 2:** Role of different cover crop types (legume, non-legume, and mixed) in nitrous oxide emissions (N_2_O).

Author	Soil-types	Vegetation	Cover crop	Cc type	Range of N_2_O emissions		Units
					Control	Cover crop	
Kallenbach et al., 2010	Coarse-loamy	Tomato	Winter legume	Legume	21.46	55.46	μg N_2_O m^–2^ h^–1^
Kallenbach et al., 2010	Coarse-loamy	Tomato	Winter legume	Legume	26.05	82.76	μg N_2_O m^–2^ h^–1^
Kallenbach et al., 2010	Coarse-loamy	Tomato	Winter legume	Legume	45.98	148.66	μg N_2_O m^–2^ h^–1^
Kallenbach et al., 2010	Coarse-loamy	Tomato	Winter legume	Legume	103.45	183.14	μg N_2_O m^–2^ h^–1^
Garland et al., 2011	Silty clay	Grape vineyard	Mix legumes	Legume	1.71 to 1.90	0.60 to 1.06	kg N_2_O-N ha^–1^ d^–1^
Mitchell et al., 2013	Clarion loam series	Maize–soybean	Rye	Non-legume	1.52 to 5.06	1.03 to 5.14	kg N_2_O-N ha^–1^ d^–1^
Sanz-Cobena et al., 2014	Silty clay loam	Maize	Barley	Non-legume	–0.26	0.02	g N_2_O-N ha^–1^ d^–1^
Sanz-Cobena et al., 2014	Silty clay loam	Maize	Rape	Non-legume	–0.26	0.72	g N_2_O-N ha^–1^ d^–1^
Sanz-Cobena et al., 2014	Silty clay loam	Maize	Vetch	Legume	–0.26	0.03	g N_2_O-N ha^–1^ d^–1^
Sanz-Cobena et al., 2014	Silty clay loam	Maize	Barley	Non-legume	0.31 to 0.50	0.93 to 3.09	g N_2_O-N ha^–1^ d^–1^
Sanz-Cobena et al., 2014	Silty clay loam	Maize	Rape	Non-legume	0.31 to 0.50	0.34 to 1.35	g N_2_O-N ha^–1^ d^–1^
Sanz-Cobena et al., 2014	Silty clay loam	Maize	Vetch	Legume	0.31 to 0.50	1.98 to 3.17	g N_2_O-N ha^–1^ d^–1^
Pramanik et al., 2014	Silt loam	Rice	Barley + hairy vetch	Mixed	0.41	0.32 to 0.37	mg m^–2^ h^–1^
Steenwerth and Belina, 2008	Loam series	Rice	Rye	Non-legume	1.51	2.33	g N_2_O-N ha^–1^ d^–1^
Steenwerth and Belina, 2008	Loam series	Rice	Trios	Legume	1.51	1.98	g N_2_O-N ha^–1^ d^–1^
Jarecki et al., 2009	Silty clay loam	Maize–soybean	Rye	Non-legume	2.10 to 8.87	3.04 to 6.37	kg N_2_O-N ha^–1^
Bavin et al., 2009	Silt loam	Soybean	Winter rye	Non-legume	0.162 to 1.79	0.27 to 1.17	nmol m^–2^ s^–1^
Abdalla et al., 2014	Sandy loam	Spring barley	Mustard	Non-legume	41.21 to 56.92	51.45 to 70.79	g N_2_O-N ha^–1^ d^–1^
Sarkodie-Addo et al., 2003	Silt loam	Maize	Rye	Non-legume	8.17 to 13.97	7.83 to 19.30	g N_2_O-N ha^–1^ d^–1^
Sarkodie-Addo et al., 2003	Silt loam	Maize	Winter wheat	Non-legume	7.51 to 22.11	6.21 to 36.96	g N_2_O-N ha^–1^ d^–1^
Negassa et al., 2015	Fine loamy	Maize	Rye	Non-legume	0.176 to 0.202	0.17 to 0.20	mg m^–2^ h^–1^
Hwang et al., 2017	Clay loam	Rice	Barley	Non-legume	0.07 to 0.1083	0.05 to 0.07	mg m^–2^ h^–1^
Hwang et al., 2017	Clay loam	Rice	Hairy vetch	Legume	0.07 to 0.1083	0.11 to 0.12	mg m^–2^ h^–1^
Hwang et al., 2017	Clay loam	Rice	Barley + hairy vetch	Mixed	0.07 to 0.1083	0.10 to 0.14	mg m^–2^ h^–1^
Abdalla et al., 2014	Sandy loam	Spring barley	Mustard	Non-legume	44.20	58.04	g N_2_O-N ha^–1^ d^–1^
Petersen et al., 2011	Loamy sandy soil	Spring barley	Fodder radish	Non-legume	44.44 to 58.60	62.59 to 97.28	g N_2_O-N ha^–1^ d^–1^
Zhao et al., 2015	Anthrosols	Rice	Rice/fava bean	Legume	0.51 to 3.69	0.20 to 1.28	μg N_2_O m^–2^ h^–1^
Zhao et al., 2015	Anthrosols	Rice	Rice/milk vetch	Legume	0.51 to 3.69	0.27 to 1.18	μg N_2_O m^–2^ h^–1^
Guardia et al., 2016	Silty clay loam	Maize	Barley	Non-legume	0.03 to 0.81	0.07 to 0.50	mg N_2_O-N m^–2^ d^–1^
Guardia et al., 2016	Silty clay loam	Maize	Vetch	Legume	0.03 to 0.81	0.04 to 1.00	mg N_2_O-N m^–2^ d^–1^
Parkin and Kaspar, 2006	Fine loamy	Maize	Rye	Non-legume	39.85 to 59.06	33.03 to 65.03	g N_2_O-N ha^–1^ d^–1^
Parkin and Kaspar, 2006	Fine loamy	Soybean	Rye	Non-legume	13.86 to 28.67	15.57 to 27.71	g N_2_O-N ha^–1^ d^–1^
Parkin et al., 2006	Clay loam	Soybean	Rye	Non-legume	0.02 to 0.37	0.04 to 0.23	g N m^–2^
Barrios-Masias et al., 2011	Silt loam	Tomato	Oat	Non-legume	0.01 to 0.24	0.01 to 0.32	mg N_2_O-N m^–2^ h^–1^
Rosecrance and Teasdale, 2000	Coarse-loamy	Maize	Cereal rye	Non-legume	0.08 to 2.04	0.10 to 0.72	ng g^–1^ d^–1^
Rosecrance and Teasdale, 2000	Coarse-loamy	Maize	Hairy vetch	Legume	0.08 to 2.04	0.59 to 10.71	ng g^–1^ d^–1^
Rosecrance and Teasdale, 2000	Coarse-loamy	Maize	Hairy vetch + cereal rye	Mixed	0.08 to 2.04	0.155 to 1.49	ng g^–1^ d^–1^
Rosecrance and Teasdale, 2000	Silt loam	Maize	Cereal rye	Non-legume	0.29 to 4.89	2.59 to 42.65	ng g^–1^ d^–1^
Rosecrance and Teasdale, 2000	Silt loam	Maize	Hairy vetch	Legume	0.29 to 4.89	3.70 to 33.70	ng g^–1^ d^–1^
Bhattacharyya et al., 2012	Sandy clay loam	Rice	*Sesbania aculeata*	Legume	3.57 to 7.63	4.61 to 25.30	μg m^–2^ h^–1^
Liebig et al., 2010	Silt loam	Wheat	Rye	Non-legume	–0.25 to 18.62	–8.26 to 24.32	μg N_2_O-N m^–2^ h^–1^
Xiong et al., 2002	Hydragric anthrosols	Early rice	Vetch	Legume	9.90 to 21.00	11.10 to 167	μg N m^–2^ h^–1^
Baggs et al., 2003	Silt loam soil	Maize	Rye	Non-legume	104 to 312	158 to 3542	g N_2_O-N ha^–1^
Baggs et al., 2003	Silt loam soil	Maize	Bean	Legume	104 to 312	113 to 2581	g N_2_O-N ha^–1^
Omonode et al., 2009	Silty clay loam	Maize	Soybean	Legume	0.02 to 651.81	0.71 to 558.64	g N_2_O-N ha^–1^ d^–1^
Dietzel et al., 2011	Howard gravelly loam	Maize	Rye	Non-legume	16.44 to 94.68	7.69 to 93.12	ng N_2_O-N cm^–2^ h^–1^
Fronning et al., 2008	Sandy loam	Maize	Rye	Non-legume	–0.44 to 46.56	0.08 to 31.01	g N_2_O-N ha^–1^ d^–1^

Cover crops are most commonly used as catch crops to mitigate nitrates leaching during fall and winter periods (Plaza-Bonilla et al., 2015). Legume CCs alone or in combination with NLCCs as a GM provide additional N for the subsequent crop (Tribouillois et al., 2015). When compared to unfertilized plots, CCs had higher mean daily N_2_O emissions but lower yearly N_2_O emissions when compared to fertilized cropping systems. CC treatments had a two- to fourfold higher potential for nitrification, mineralization, and denitrification than conventional cultivation. Thus, CCs improved the soil capacity to support higher MBN, potential N mineralization, and microbiological functions of nitrification and denitrification. Kerri and Belina (2008) found that total soil C content was 40–50% higher in soils under five consecutive years of annual CCs than continuously cultivated soil. Cover crops also influence the quality of soil water by rising transpiration rate than bare soil. In general, existing studies show little effect of CCs (LCCs or NLCCs) on N_2_O emissions, particularly when results are integrated on the basis of residues quality and quantity (Muhammad et al., 2019). According to a meta-analysis, the effect of CCs on N_2_O emissions is mostly influenced by the residues C:N ratio, climatic condition, and residues management practices (Muhammad et al., 2019). Similarly, another meta-analysis demonstrated that the introduction of CCs residues into soil often leads to a short-term increase in N_2_O emissions, especially for LCCs (Basche et al., 2014).

Higher precipitations have a deleterious effect on N_2_O emissions from CCs fields. CC residue incorporation through tillage has a significant impact on soil structure, soil water dynamics, soil nutrients, and organic residues, which may have an impact on crop production (Gregorich et al., 2008; Negassa et al., 2015). Soil N_2_O is mainly produced by soil microorganisms through nitrification and denitrification. Similarly, farmland cultivation measures also affect the soil N_2_O emission process by affecting soil temperature, humidity, and nutrient status (Sanz-Cobena et al., 2012). A previous study reported that CCs mulching significantly reduces N_2_O emissions (Fiorini et al., 2020), whereas some researchers have found that soil N_2_O emissions significantly increased with CCs incorporation (Snapp and Borden, 2005; Tang et al., 2015). Compared to saturated moisture, higher soil moisture content (45–75%) had significantly higher N_2_O emissions. Nitrous oxide emissions increase with higher soil water content and gradually decrease after reaching saturated water content (Sheppard et al., 2013).

### Methane Emissions

Cold-resistant legumes like Chinese milk vetch and hairy vetch and NLCCs like rye and barley could be the only option for winter CCs in temperate countries with cold and dry weather during winter seasons. Vetch is considered the most common GM in rice fields due to its high N-fixing capacity to adapt to harsh winter conditions and better growth in wet paddy soil. Hwang et al. (2017) demonstrated that hairy vetch and barley mixtures as GM are favored in rice paddy soil because of their higher biomass productivity than sole vetch or barley crops and stronger resistance to winter drought (Ahmad et al., 2022). The addition of NLCCs (barley or rye) would not efficiently increase rice production due to the high C:N ratio and slow mineralization process of crop residues. The combination of barley and hairy vetch therefore reduce the C:N ratio below 20, which support the mineralization of organic substrates in soil (Marton et al., 2014). During rice cultivation, CCs biomass application as GM increased soil C balance by 39–142% over NCCs and increased the seasonal net global warming potential by 3.2–5.7 times due to significantly higher CH_4_ emissions under flooded soil conditions.

As shown in Table 3, the CH_4_ emission ranges from 73.52–83.08 mg CH_4_ m^–2^ h^–1^ in milk vetch CCs, which is significantly higher than control (0.74–26 mg CH_4_ m^–2^ h^–1^), and rye CCs (1.11–80.14 mg CH_4_ m^–2^ h^–1^) in rice paddy soil (Sang et al., 2012). Most lowlands are under entirely irrigated or rain-fed conditions, and their cultivation serves as an important source of CH_4_ emissions (Zschornack et al., 2016). The average global CH_4_ emissions from rice paddy fields are reported to be around 11% of the overall anthropogenic CH_4_ emissions (Yan et al., 2009). It was stated previously that the production of rice, which was 473 million tons in 1990, needs to be increased by 39.43% in 2020 to meet the world population food demand and that anthropogenic CH_4_ emissions have been raised by 40–50% (Lee et al., 2010). Since CH_4_ is primarily produced under strictly anaerobic conditions, it might be because of the decay of organic matter through archaeal methanogens (Chandio et al., 2020). Similarly, the introduction of organic materials such as GM into flooded rice fields will promote CH_4_ emissions by methanogens with readily available C. Agricultural Mediterranean soils produce significant CH_4_ emissions through methanogens in flooded crops (e.g., rice), which represent 6% of the total agricultural production (Sanz-Cobena et al., 2017). Methane emissions mainly depend on residue incorporation, and the CH_4_ emissions increase from rice paddy soil by 100–500 kg CH_4_ ha^–1^ year^–1^ when the straw is added at the rate of 0–7 tha^–1^ (Sanchis et al., 2012). Improved crop production approaches are likely to clash with the extenuation of CH_4_ emissions (Yan et al., 2009).

**TABLE 3:** Role of different cover crop types (legume, non-legume, and mixed) in soil methane emissions (CH_4_).

Author	Soil-types	Vegetation	Cover Crop	CC type	Range of CH_4_ emissions		Units
					Control	Cover crop	
Sang et al., 2012	Fine silty	Rice	Rye	Non-legume	0.74 to 25	1.11 to 80.14	mg CH_4_ m^–2^ h^–1^
Sang et al., 2012	Fine silty	Rice	Milk vetch	Legume	0.74 to 26	73.52 to 83.08	mg CH_4_ m^–2^ h^–1^
Sanz-Cobena et al., 2014	Silty clay loam	Maize	Barley	Non-legume	0.08 to 5.05	0.084 to 5.96	g CH_4_-C ha^–1^ d^–1^
Bavin et al., 2009	Silt loam	Soybean	Winter rye	Non-legume	0.05 to 0.27	0.03 to 0.25	nmol m^–2^s^–1^
Changhoon et al., 2010	Fine silty mixed mesic,	Rice	Chinese milk vetch	Legume	8.99 to 85.39	5.99 to 316.11	mg m^–2^ h^–1^
Haque et al., 2013	Silt loam	Rice	Barley + Vetch	Mixed	3.75 to 13.13	7.15 to 180.17	mg m^–2^ h^–1^
Peyrard et al., 2016	Calcareous clay	Wheat-sunflower	Barley	Non-legume	0.09 to 1.04	0.51 to 1.55	mg m^–2^ h^–1^
Peyrard et al., 2016	Calcareous clay	Wheat-sunflower	Hairy Vetch	Legume	0.09 to 1.04	0.16 to 3.17	mg m^–2^ h^–1^
Peyrard et al., 2016	Calcareous clay	Wheat-sunflower	Barley + Vetch	Mixed	0.09 to 1.04	0.21 to 1.96	mg m^–2^ h^–1^
Peyrard et al., 2016	Calcareous clay	Wheat-sunflower	Barley	Non-legume	2.01 to 19.15	1.00 to 172.37	mg m^–2^ h^–1^
Peyrard et al., 2016	Calcareous clay	Wheat-sunflower	Hairy Vetch	Legume	2.01 to 19.15	1.00 to 50.40	mg m^–2^ h^–1^
Peyrard et al., 2016	Calcareous clay	Wheat-sunflower	Barley + Vetch	Mixed	2.01 to 19.15	2.01 to 186.49	mg m^–2^ h^–1^
Hwang et al., 2017	Clay loam	Rice	Barley	Non-legume	–1.60 to 25	18 to 170	mg m^–2^ h^–1^
Hwang et al., 2017	Clay loam	Rice	Hairy Vetch	Legume	–1.60 to 25	–1 to 130	mg m^–2^ h^–1^
Hwang et al., 2017	Clay loam	Rice	Barley + Vetch	Mixed	–1.60 to 25	1 to 190	mg m^–2^ h^–1^
Abdalla et al., 2014	Sandy loam	Spring barley	Mustard	Non-legume	0.05 to 0.50	0.01 to 0.47	g CH_4_-C h^–1^ d^–1^
Bhattacharyya et al., 2012	Sandy clay loam	Rice	Sesbania aculeata	Legume	1.92 to 3.02	2.81 to 8.64	mg m^–2^ h^–1^
Fronning et al., 2008	Sandy loam	Maize	Rye	Non-legume	1.20 to 1.50	1.10 to 50	g CH_4_-C h^–1^ d^–1^

## Residues C:N Ratios and Greenhouse Gas Emission

Cover crop residue C:N ratios have a direct impact on soil GHG (CO_2_, N_2_O, and CH_4_) emissions. Results showed that residues with a high C:N ratio significantly increase CO_2_ and CH_4_ fluxes while decreasing N_2_O flux. These results are in line with the finding of earlier researchers (Muhammad et al., 2019), who documented that CO_2_ emissions are positively and N_2_O emissions are negatively affected by increasing the residues C:N ratios. Huang et al. (2002) reported that seasonal N_2_O emission from wheat-cultivated soil was negatively correlated with increasing soil C:N ratio. The N_2_O/NO_3_^–^ ratio and N_2_O emission rate increased with decreasing C:N ratio in organic amendments in a well-aerated soil (Melling et al., 2007). Reddy and Crohn (2014) demonstrated that the rate of N_2_O production is partially controlled by C susceptible through the mineralization process. Compared with wheat monoculture, wheat-chickpea crop rotation showed a C sequestration rate of 0.53 Mg C ha^–1^ y^–1^ during 20 years (López-Bellido et al., 2010), which could be related to the fluxes of CO_2_. Huang et al. (2004) found that the root decay content was strongly negatively correlated with increasing the root residue C:N ratio. Similarly, Khalil et al. (2002) reported that N_2_O production was increased by decreasing the C:N ratio of different organic materials. In addition, Kato et al. (2011) found a strongly negative correlation between the mean annual N_2_O emission and C:N ratio of tropical rain forest soils. Residues with lower C:N decomposed more rapidly, might provide a greater opportunity for producing more dissolved organic C, hence resulting in higher N_2_O emissions.

### Low C:N Ratio Residues

Cover crops develop resistance against pests, weeds, and potential environmental degradation, improve soil quality, and thus increase the production of subsequent cash crops (Neely et al., 2018). Leguminous CCs release N into the soil and thus enhance crop yield (Holman et al., 2018). Lentil (*Lens culinaris Medik*), field pea (*Pisum sativum L*.), and faba bean (*Vicia faba L*.) were used as LCCs for potential N fixation in rotation with cash crops. The biennial clover (*Melilotus officinalis L*.) has advantages over annual legumes because of its low seed costs, a strong competitor to weeds, and high productivity in biomass production and N fixation in semi-arid environments (Ravenel et al., 2015). Intercropping LCCs with winter cereals may have fixed atmospheric N_2_, but after winter cereal harvest, they may show maximum growth and N fixation. Cover crops such as alfalfa (*Medicago sativa L*.) and red clover (*Trifolium pratense L*.) had higher production of biomass and added N to winter wheat crops due to their faster decomposition rate (Chen et al., 2006). The research revealed that alfalfa and red clover as relay crops and lentil and chickling vetch (*Lathyrus sativus L*.) as double crops were productive legumes in a cereal cropping system in winter (Martens and Entz, 2001). It has been shown that alfalfa and red clover can fix significant amounts of N without adversely affecting the winter wheat yield in moderate to high rainfall regions (Martens and Entz, 2001), while the incorporation of these crops into the soil increases soil health due to mineralization through soil microorganisms (Li et al., 2019). Similarly, alfalfa and red clover showed potential for N fixation in moderate to high rainfall areas without adversely affecting the yield of winter wheat (Martens et al., 2001).

### High C:N Ratio Residue

The CCs are selected based on N-fixing capacity, biomass quality (C:N ratios), and quantities in many agricultural systems. However, there is insufficient evidence to suggest that crops increase soil fertility, crop yield, and soil microbial populations (Blesh, 2018). Non-legume CCs are also the best choice for spring forage because of their regrowth behavior during the growth stage as compared to small grain cereals (White and Weil, 2010). CCs during the following seasons decrease the soil water and wind erosion due to covering soil surface (Kaspar et al., 2001), and rye CCs help to sustain higher SOM (Kaspar et al., 2006). Researchers proposed that CCs decrease soil bulk density and increase water retention potential, soil microbial processes, and porosity of soils (Villamil et al., 2006). However, other researchers reported that CCs have either improved or have no effect on soil properties (Mupambwa, 2012; Mupambwa and Wakindiki, 2012). Soil response to agronomic methods is also influenced by soil and environmental conditions (Bescansa et al., 2006) in addition to CCs management. The introduction of rye CCs conserved soil resources and had environmental benefits immediately and long-term economic benefits during the cropping system (Igos et al., 2016).

### Optimum C:N Ratio Residues or Mixed Residues

Mixed CCs are an important management method used extensively to increase SOM and subsequently increase cash crop productivity (Sainju and Singh, 2008). Growing CCs can provide physical protection to the soil by reducing the impact of rainfall and also improve soil structure and aggregation (Tang et al., 2017), and similarly improve soil microorganisms (Araujo et al., 2019). Morales Salmeron et al. (2019) found that soil structure and fertility are improved by the presence of legumes and legume-grain mixtures in row crop systems. In addition, the residues of the main crop rotated with a potato crop are supposed to affect the accumulation of soil N and C over time. Planting CCs mixtures (multi-species) could be a viable solution to enhance the ecological stability, microbial diversity, and resilience of CCs communities, which contribute to higher and consistent productivity. The production benefits of multi-species plant communities include the potential for increased efficiency in resource use and crop yields (Tosti et al., 2012).

## Termination Method to Mitigate Greenhouse Gas Emissions

The goal of cultivation and the availability of machinery are the two main factors that mitigate GHG emissions. The CCs are terminated through incorporation, surface mulch, and/or removal, as well as using herbicide for killing or improving the productivity of the following cash crop. The influence of CCs variety, termination timing, and termination method on mulch, weed cover, and soil nitrate in organic systems plays a vital role in GHG emissions.

### Residues Incorporation

**FIGURE 7:**
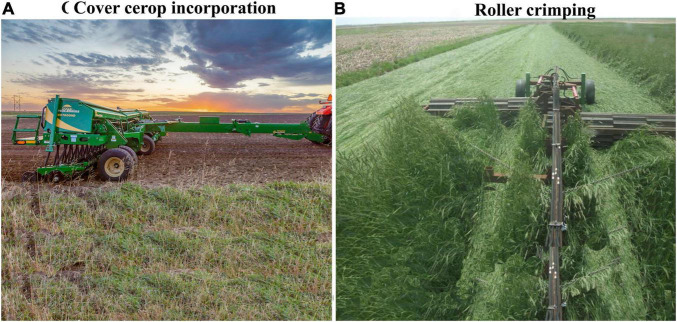
**(A,B)** Examples of cover cropping termination methods (incorporation, mulching, and removing), and the concept of cover crop residues biomass and soil microbes interaction.

A traditional termination method involves using a fixed machine-driven disk to NCCs and CCs plot (Figure 7A) to incorporate residues into the soil (Jani et al., 2016; Rojas et al., 2018). Termination of CCs through disking results in rapid residue decomposition and release of nutrients due to close contact with soil microorganisms. As a result, microbial decomposition has been facilitated by better residue soil interaction and higher levels of soil oxygen. Cover crop incorporating through disking improved the total bacteria population while decreasing or stabilizing the populations of fungi and actinomycetes (Elfstrand et al., 2007). The incorporation of barley and rape CCs residue increased the soil respiration by 21 and 28%, respectively. The CH_4_ emissions were decreased with CCs incorporation with mean values of –0.12 and –0.10 kg CH_4_–C ha^–1^ for plots with and without CCs, respectively (Table 3; Sanz-Cobena et al., 2014). Fungi abundance and reduction in the disked soils could be due to high lignin materials, increased soil disturbance, and reduced soil moisture (Elfstrand et al., 2007). Incorporating CCs residue into the soil increased CO_2_ and N_2_O emissions compared to residue placed on the soil surface because of increased contact with soil microorganisms.

Tillage practices expose more residue to microorganisms, which enhances aeration and microbial activity, resulting in higher CO_2_ and N_2_O emissions (Alluvione et al., 2010). Several studies have found that incorporating CC residues into the soil increases N_2_O emissions when compared to surface placement (Xiong et al., 2002; Basche et al., 2014; Muhammad et al., 2021b). Further research is needed to examine the impact of disking on CC residues breakdown, retention of soil water content, OM, dynamics of N releases, and subsequent cash crops. CCs with a high C:N ratio, such as rye, stimulated more CH_4_ emissions than CCs with a low C:N ratio per grain yield, but LCCs vetch was more effective in improving the rice growth and yield (Table 3). To improve soil properties and rice productivity while limiting CH_4_ release, it may be more beneficial to use a low C:N ratio, such as milk vetch, as a CC than a high C:N ratio, such as rye (Sang et al., 2012).

### Residues Mulching

Using a rotary or flail mower to terminate CCs cuts the residue into slices and shreds the residue on the soil surface (Rose et al., 2016). In early fall, the soil microbial biomass was 150 mg C kg^–1^ soil, which is 50% higher than in late spring (100 mg C kg^–1^ soil; Zibilske and Makus, 2009). Mowing rye at the vegetative growth stage resulted in regrowth, which eventually resulted in water and nutrient competition with subsequent cash crops (Kornecki et al., 2009). The termination of CCs with rotating mowers can be difficult due to the irregular size and dispersal of plant remains. Compared to the arable weed community present in NCCs plots, LCCs increased total PLFA concentration by 5.37 nmol g^–1^ and 10.20 nmol g^–1^ in the fall and spring (Finney et al., 2017). Hairy vetch was positively associated with non-AMF (*Vicia villosa* L.). The associations between CCs and microbial groups were found in monocultures and in multispecies CC mixtures (Finney et al., 2017). In comparison to CCs incorporation, CCs termination with a flail mower may be important in controlling soil nutrient transformations and SOM maintenance in subtropical climates, which regulate soil temperature and moisture at more suitable levels for microbial activity and residue decomposition (Zibilske and Makus, 2009; Khan et al., 2021). Moreover, another study found that flail-mowing is difficult to achieve uniform mulching, allowing weeds to emerge via thin openings (Teasdale et al., 2007; Wegner et al., 2018).

Mechanical mowing of rye, mustard, and hairy vetch mixture improved N mineralization. Compared to the NCCs treatment, white mustard (*Sinapis alba* L.), Lacy phacelia (*Phacelia tanacetifolia Benth*. LP), and hairy vetch (*Vicia villosa Roth*) mulching increased microbial biomass C by 38, 80, and 44%, respectively. These increases could be due to the increase in soil C and N by 19 and 44% in white mustard, 6 and 2% in Lacy phacelia, and 10 and 13% in hairy vetch, respectively, when compared to NCCs (Marinari et al., 2015). Several studies have found that incorporating CC residues increases N_2_O emissions when compared to surface placement (Basche et al., 2014; Muhammad et al., 2019). The CCs residue removal had no effect on N_2_O emissions when compared to NCCs but resulted in higher CO_2_ emissions because of CCs roots in the soil that were not removed. Only the above-ground biomass of CCs was removed from the soil for animal forage in the residue removal treatment.

Timing is the most crucial factor when operating a roller-crimper for CCs termination. Termination of grass CCs should occur once flowering has begun (Figure 7B), while LCCs should be terminated after pods formation. The CCs will not be terminated successfully if it is performed too late (McMechan et al., 2021; Werle et al., 2021). The use of a roller-crimper is one of the best ways to suppress weeds with CCs residue (Mirsky et al., 2009). To avoid competing with cash crops for water and nutrients, effective termination of CCs with a roller-crimper is critical (Bavougian et al., 2019). It has been demonstrated that crimping LCCs with roller crimped produce 10 to 217 kg N ha^–1^ reliant on CCs species and killing times (Parr et al., 2014), and soil inorganic N peaks after 4 to 6 weeks of roller crimping (Parr et al., 2014). Further study is required to discover how mulching and removing the CCs termination method affects soil C, N release, and soil microbial diversity in agricultural ecosystems.

### Residue Removal With Herbicide Application

Non-selective herbicides are commonly used for terminating the CCs because they are effective at all growth phases (Clark et al., 2007). These have low application costs and the ability to terminate CCs on a large scale within a short period of time (Hay et al., 2019; Farooq et al., 2022). As the role of herbicides in non-target species has increased, along with their negative impact on these species, there is an increased desire to learn about their impact on plant nutrition and nutrient cycling (Cornelius and Bradley, 2017). Previous studies have demonstrated that glyphosate has no impact on soil microorganisms diversity and activity (Weaver et al., 2007; Lane et al., 2012), although, after one week of glyphosate treatment, a significant drop in total microbial biomass in soybean rhizosphere soil was detected (Lane et al., 2012). Selection of herbicide with residual soil activity when planning to use CCs again in the fall must be done carefully to avoid impacts on CCs species establishment. Glyphosate should be applied when temperatures reach 55°F during the day and 40°F during the night. Applying glyphosate before the boot stage will help to improve effective cereal rye. Improved spray coverage will increase the efficacy of contact herbicides such as paraquat (Gramoxone) and glufosinate (Liberty, Cheetah, and Scout; Werle et al., 2021). During the spring thaw until the end of rye, CO_2_ emissions were higher in green fallow than in chemical fallow (*P* = 0.0071). The atmospheric CH_4_ uptake was the dominant exchange process, and it was significantly (*P* = 0.0124) higher under chemical fallow (2.7 g CH_4_-C ha^–1^ d^–1^) than under green fallow (1.5 g CH_4_-C ha^–1^ d^–1^). Cumulative CO_2_, CH_4_, and N_2_O emissions did not differ between the chemical and green-fallow phases (*P* = 0.1293, 0.2629, and 0.9979, respectively) during the 19-month period (Liebig et al., 2010).

## Conclusion

The effect of CC types, biomass, and residue C:N ratios on soil GHGs emissions, soil microbial biomass, and community was investigated in this study. Compared to NCCs, the CCs increased microbial biomass and community abundance due to additional organic matter input. A higher fungi/bacteria ratio with CCs suggests that CCs have a greater impact on fungi than bacteria. The LCCs had lower actinomycete levels but a higher MBC/MBN ratio than the NLCCs. The benefits of CCs on soil microbial biomass were reduced when mixed LCCs and NLCCs were used instead of LCCs or NLCCs alone. Carbon dioxide and N_2_O emissions vary according to CCs species, biomass residue quality and quantity, and method of residue placement in the soil. When compared to NCCs, CCs increased CO_2_ emissions. Legume CCs emitted more N_2_O than NLCCs or mixed CCs. Increased CCs biomass resulted in higher CO_2_ emissions but lower N_2_O emissions. The increases in N_2_O emissions can cause changes in global warming potential, thus affecting the C sinks (soil organic C input via plants) and losses (CO_2_ emissions/mineralization). The LCC and NLCCs combined application as CCs reduced the GHG emissions and improved soil health and crop yields. Although CCs increase CO_2_ emissions compared to NCCs, they have positive effects on soil and C sequestration and other known soil health and environmental quality parameters. Further studies are needed to clarify the effect of CCs biomass rates and residues quality (C:N ratios) on soil microbial community structure and abundance and their influence on soil GHG emissions.

## Author Contributions

IM, XZ, and JW did the conceptualization. IM and SAh performed the methodology. IM and SF investigated the data. XZ carried out the resources. IM carried out the data curation and wrote the original draft of the manuscript. SAl, XZ, and JW wrote, reviewed, and edited the manuscript. XZ supervised the data. All authors have read and agreed to the published version of the manuscript.

## Conflict of Interest

The authors declare that the research was conducted in the absence of any commercial or financial relationships that could be construed as a potential conflict of interest.

## Publisher’s Note

All claims expressed in this article are solely those of the authors and do not necessarily represent those of their affiliated organizations, or those of the publisher, the editors and the reviewers. Any product that may be evaluated in this article, or claim that may be made by its manufacturer, is not guaranteed or endorsed by the publisher.
